# A phase II trial of dose-reduced *nab*-paclitaxel for patients with previously treated, advanced or recurrent gastric cancer (OGSG 1302)

**DOI:** 10.1007/s10147-020-01768-w

**Published:** 2020-09-14

**Authors:** Shigeyuki Tamura, Hirokazu Taniguchi, Kazuhiro Nishikawa, Hiroshi Imamura, Junya Fujita, Atsushi Takeno, Jin Matsuyama, Yutaka Kimura, Junji Kawada, Motohiro Hirao, Masashi Hirota, Kazumasa Fujitani, Yukinori Kurokawa, Daisuke Sakai, Hisato Kawakami, Toshio Shimokawa, Taroh Satoh

**Affiliations:** 1 Department of Surgery, Yao Municipal Hospital, 1-3-1, Ryuge, Yao, Osaka 581-0069 Japan; 2 Department of Gastroenterological Surgery, Osaka Saiseikai Senri Hospital, Suita, Osaka Japan; 3 Department of Surgery, National Hospital Organization Osaka National Hospital, Osaka, Japan; 4 Department of Surgery, Toyonaka Municipal Hospital, Toyonaka, Osaka Japan; 5 Department of Surgery, Sakai City Medical Center, Sakai, Osaka Japan; 6 Department of Surgery, Kansai Rosai Hospital, Amagasaki, Hyogo Japan; 7 Department of Gastroenterological Surgery, Higashiosaka City Medical Center, Higashiosaka, Osaka Japan; 8 Department of Surgery, Kindai University Hospital, Sayama, Osaka Japan; 9 Department of Surgery, Osaka General Medical Center, Osaka, Japan; 10 Department of Gastroenterological Surgery, Osaka University Graduate School of Medicine, Suita, Osaka Japan; 11 Department of Frontier Science for Cancer and Chemotherapy, Osaka University Graduate School of Medicine, Suita, Osaka Japan; 12 Department of Medical Oncology, Faculty of Medicine, Kindai University, Sayama, Osaka Japan; 13 Clinical Study Support Center, Wakayama Medical University, Wakayama, Japan

**Keywords:** Advanced gastric cancer, Recurrent gastric cancer, *Nab*-paclitaxel, Paclitaxel

## Abstract

**Background:**

For unresectable or recurrent advanced gastric adenocarcinoma (AGC), tri-weekly administration of nanoparticle albumin-bound paclitaxel (*nab*-PTX) at 260 mg/m^2^ achieved a response rate of 27.8% in a phase II trial in Japan. However, frequent neutropenia and peripheral neuropathy limit its use in clinical settings. We, thus, conducted a single-arm phase II trial to investigate the efficacy and safety of a reduced dose (220 mg/m^2^) of tri-weekly *nab*-PTX.

**Methods:**

Eligible patients included those with AGC and ECOG performance status of 0–2 who had received one or more prior chemotherapy containing fluoropyrimidine regimens. A reduced dose of *nab*-PTX (220 mg/m^2^) was administered tri-weekly. The primary endpoint was response rate (RR). Secondary endpoints were overall survival (OS), progression-free survival (PFS), disease-control rate (DCR), incidence of adverse events, relative dose intensity (RDI) and proportion of patients receiving subsequent chemotherapy.

**Results:**

Among 33 patients enrolled, 32 were treated with protocol therapy. RR was 3.1% [95% confidence interval (CI), 0–16.2%], which did not reach the protocol-specified threshold (*p* = 0.966). DCR was 37.5% (95% CI, 21.1–56.3%). Median OS and PFS were 6.3 (95% CI, 4.4–14.2) and 2.2 (95% CI, 1.8–3.1) months, respectively. RDI was 97.8%. Twenty (62.5%) patients received subsequent chemotherapy. Toxicity was relatively mild with the most common grade ≥ 3 adverse events being neutropenia (38%), anemia (13%), fatigue (19%), anorexia (16%), and peripheral neuropathy (13%).

**Conclusion:**

Tri-weekly *nab*-PTX with a reduced dose (220 mg/m^2^) is not recommended for AGC in a second-line or later setting, despite demonstrating less toxicity than at 260 mg/m^2^.

*Clinical trial registration*

The OGSG1302 trial was registered with UMIN-CTR as UMIN000000714.

## Introduction

Combination of chemotherapy with platinum agents and fluoropyrimidine has been regarded as the standard of care in a first-line setting for unresectable or recurrent advanced gastric adenocarcinoma (AGC) [1], to which trastuzumab, an anti-HER2 monoclonal antibody, is added in HER2-positive cases [2, 3]. Recently, oral fluoropyrimidine including S-1 and capecitabine, has generally been utilized instead of infusional 5-FU because of their convenience and tolerability [4–6]. The combination of S-1 and cisplatin is now accepted as the standard regimen for first-line chemotherapy for patients with AGC in Japan, based on the result of the SPIRITS trial [4].

For second-line chemotherapy, until weekly solvent-based paclitaxel (*sb*-PTX) plus ramucirumab, an anti-VEGFR antibody, demonstrated superiority over *sb*-PTX alone [7], *sb*-PTX alone was widely utilized in this setting based on a phase III study in which *sb*-PTX showed comparable efficacy to CPT-11 with less toxicity [8]. This was also supported by promising results of several phase II studies, yielding overall response rates (RRs) that ranged from 16 to 27% and overall survival (OS) times of 5–11 months [8–12]. However, *sb*-PXT can cause hypersensitivity and anaphylactic reactions in certain patients, mostly because of polyethoxylated castor oil contained in it [13]. To use this drug safely, premedication with steroids and histamine H-2 blockers is generally required. Moreover, *sb*-PTX contains alcohol. These factors limit the general use of *sb*-PTX in a certain subset of AGC patients.

Nanoparticle albumin-bound paclitaxel (*nab*-PXT) is a novel, biologically interactive, nanometer-size albumin-bound paclitaxel particle initially developed to avoid the toxicities associated with polyethoxylated castor oil. It can be administered as a high dose of paclitaxel without premedication with steroids and histamine H-2 blockers. Furthermore, *nab*-PTX can be administered in only 30 min and used safely for alcohol-intolerant patients [14]. In clinical trials for metastatic breast cancer (MBC) as well as non-small-cell lung cancer, *nab*-PTX demonstrated efficacy equivalent to or exceeding that of *sb*-PTX [15–17]. In patients with AGC, a phase II trial in Japan showed the efficacy of tri-weekly *nab*-PTX at 260 mg/m^2^ without anti-allergic premedication, with an overall RR, the primary endpoint of this study, of 27.8% [15/54; 95% confidence interval (CI), 16.5–41.6%] and the median progression-free survival (PFS) and OS being 2.9 (95% CI, 2.4–3.6) and 9.2 (95% CI, 6.9–11.4) months, respectively [18]. However, relatively high toxicity was indicated, with the most common grade 3/4 toxicities being neutropenia (49.1%), leucopenia (20.0%), lymphopenia (10.9%) and chemotherapy-induced peripheral neuropathy (CIPN) (23.6%) [18]. Against this background, the optimal dosing that can minimize toxicity without sacrificing anticancer efficacy remains to be established.

A phase I trial of *nab*-PTX in patients with advanced solid tumors determined the maximum tolerated dose to be 300 mg/m^2^ [19]. In MBC, the dose of *nab*-PTX was initially set as 300 mg/m^2^ [20], and then deduced to 260 mg/m^2^ in the subsequent phase III Ca012 trial, where tri-weekly *nab*-PTX demonstrated significantly superior RR as well as a longer time to progression compared to the conventional *sb*-PTX at a dose of 175 mg/m^2^ [15]. This study also identified grade 3 or higher CIPN as a considerable adverse event of *nab*-PTX [15]. To reduce such toxicity, low-dose tri-weekly *nab*-PTX (160–175 mg/m^2^) was examined in several phase II studies for MBC, showing good overall RR (23–39.5%) without CIPN of grade 3 or higher [21, 22].

These results reveal the need to develop a low-dose *nab*-PTX regimen in AGC. To this end, we conducted a phase II trial to evaluate the efficacy and safety of low-dose tri-weekly *nab*-PTX (220 mg/m^2^) in AGC patients in second-line or later setting.

## Patients and methods

### Study objectives and design

This study was conducted in accordance with the international ethical recommendations stated in the Declaration of Helsinki. The protocol was approved by the institutional ethics committees of each participating hospital and registered in the University Hospital Medical Information Network (UMIN) database (ID000000714). Written informed consent was obtained from each patient before enrollment.

This was a non-randomized, multicenter phase II study for patients with AGC for whom more than one regimen including fluorinated pyrimidine antineoplastic agents had failed. The primary endpoint was RR, and the secondary endpoints were OS, PFS, time to treatment failure (TTF), disease-control rate (DCR), safety, relative dose intensity and proportion of patients who received subsequent therapy. This trial was carried out in accordance with the Japanese Classification of Gastric Carcinoma of the 14th edition from the Japanese Gastric Cancer Association.

### Eligibility criteria

The eligibility criteria of this study were as follows; (1) histologically confirmed unresectable or recurrent gastric or esophagogastric junction adenocarcinoma; (2) history of failure of one or more prior chemotherapy containing fluoropyrimidine regimens for HER2-negative cases or both fluoropyrimidine and Trastuzumab for HER2-positive cases; (3) age 20–80 years; (4) Eastern Cooperative Oncology Group (ECOG) performance status (PS) of 0–2; (5) one measurable lesion according to RECIST ver. 1.1 criteria as determined via computed tomography (CT) within 4 weeks before enrollment; (6) no previous treatment with PTX; (7) adequate organ function, including leukocyte count under 12,000 mm^3^, neutrophil count over 2,000 mm^3^, platelet count over 100,000 mm^3^, hemoglobin level over 9.0 g/dl, serum bilirubin level under 1.5 mg/dl, aspartate aminotransferase (AST) and alanine aminotransferase (ALT) levels of under 100 or 200 IU/L of patients with liver metastasis, a serum creatinine level under 1.5 mg/dl; (8) expected to survive for at least 90 days from the date of registration; and (9) cases with the provision of informed consent.

Exclusion criteria were as follows: (1) with a history of severe drug sensitivity; (2) with infection or suspected infection with a fever over 38.0 °C; (3) serious complications, such as interstitial pneumonia or lung fibrosis, uncontrolled diabetes, or renal or hepatic failure; (4) suffering more than four bouts of diarrhea; (5) a history or complication of heart disease, for example, congestive heart failure, myocardial infarction, ischemic heart disease requiring treatment, arrhythmia or valvular disease; (6) active double cancer; (7) peripheral neuropathy over grade 2; (8) difficulty enrolling due to a psychiatric or neurological disorders; (9) brain metastasis; (10) positivity for HBs antigen or HCV antibody, and (11) the a presence of any other condition that would make the treatment unsafe.

Written informed consent was obtained from each patient before enrollment and the protocol was approved by the institutional ethics committee of each participating centers.

### Study design

#### Treatment

*nab*-PTX was administered intravenously on an outpatient basis by a 30-min infusion at a dose of 220 mg/m^2^ on day 1 of each 21-day cycle. No premedication, such as steroid or antihistamine premedication, was administered. Treatment was continued until disease progression, unacceptable toxicity, or consent withdrawal.

Two dose-reduction levels (level 1, 180 mg/m^2^ and level 2, 150 mg/m^2^) and one dose escalation level (260 mg/m^2^) were implemented under the dose-reduction or escalation criteria: if the number of neutrophils was 1500/mm^3^ or more after the administration of 220 mg/m^2^ *nab*-PTX in the previous course and the dose-reduction criteria were not violated, the dose of *nab*-PTX could be increased up to 260 mg/m^2^ in the next course.

### Follow-up

Patients underwent hematological tests and assessments of clinical symptoms at least once during each course of chemotherapy. However, in the first course, hematological tests were conducted on the 1st, 8th and 15th day. The severity of adverse drug reactions was judged in accordance with the National Cancer Institute Common Terminology Criteria for Adverse Events, version 3.0. Thoracoabdominal CT scans were repeated at least every 6 weeks (± 2 weeks) after treatment initiation and at the end of the treatment in this study. The objective disease status was assessed in accordance with the RECIST guidelines, version1.1. An independent review board organized by the Osaka Gastrointestinal Cancer Chemotherapy Study Group (OGSG) objectively identified treatment responses and drug-related adverse events.

### Statistical analysis

RR was reported to be 27.8% (95% CI: 16.5–41.3) in a phase II study of nab-PTX (260 mg/m^2^, q3w) with almost the same objective as the present study [18] and 23% in a phase II study of PTX (210 mg/m^2^; 95% CI: 13–36%) [9]. The calculation of the sample size for the study was based on an expected response rate of 25% and a threshold response rate of 10%, using a one-sided alpha error of 0.05 and statistical power of 80%. The planned sample size was 35 patients, allowing for four patients dropping out.

The analysis focused on patients who were enrolled in this study and received at least one course of *nab*-PTX treatment. Background data were summarized as frequency with proportion for categorical variables, and median with range for continuous variables. The response rate was evaluated using exact binomial test. Confidence intervals of response rate and disease-control rate were estimated by the Clopper–Pearson method. OS, PFS and TTF were estimated using the Kaplan–Meier method and the 95% CIs for survival rate were calculated using Greenwood’s formula. *p* values less than 0.05 were considered statistically significant. All statistical analyses were performed with S-plus version 3.6.1 (R Foundation for Statistical Computing, Vienna Austria).

## Results

Between April 2014 and December 2018, 33 patients with AGC and ECOG PS of 0–2 who had received one or more prior chemotherapy containing fluoropyrimidine regimens were enrolled from 10 institutions in Japan. As one patient withdrew consent before the initial treatment, 32 patients received the study treatment and were evaluated for clinical response and safety. The patients’ characteristics are listed in Table 1. Twenty-seven patients were male (84.4%) and the median age was 70 years (range 48–82). Most of the patients had an ECOG PS of 0 or 1, whereas ECOG PS 2 was seen in two patients (6%). Twenty patients involved advanced cases and 12 involved relapse. The stages at initial treatment of the relapse cases were stage II for 1 patient, stage III for 9, and stage IV for 2. Twenty-three patients (72%) were enrolled as second-line treatment and nine patients (28%) as third-line treatment.

**Table 1:** Baseline characteristics of the patients

	*n* = 32
Gender	
Male	27 (84%)
Female	5 (16%)
Age	
Median (range)	70 (48**–**82)
ECOG performance status	
0	18 (56%)
1	12 (38%)
2	2 (6%)
Body mass index	
Median (range)	20.0 (16.0**–**27.0)
Diagnosis	
Advanced	20 (63%)
Relapse	12 (38%)
Previous gastrectomy	
Yes	5 (16%)
No	27 (84%)
Metastatic site	
None	10 (31%)^a^
Liver	7 (22%)
Lymph nodes	4 (13%)
Liver + peritoneum	3 (9%)
Peritoneum	7 (22%)
Liver + lymph node	1 (3%)
Number of previous chemotherapy regimens	
1	23 (72%)
2	9 (28%)
Previous systemic anticancer agents	
S-1	25 (78%)
CDDP	18 (56%)
Capecitabine	7 (22%)
CPT-11	6 (19%)
Oxaliplatin	6 (19%)
Docetaxel	2 (6%)
Other^b^	4 (13%)

Data are* n* (%)

^a^All 10 patients were relapsed cases.

^b^Other included two patients with UFT (tegafur + uracil), one with TAS118 (S-1 + leucovorin) and one with trastuzumab.

### Efficacy

**Fig. 1:**
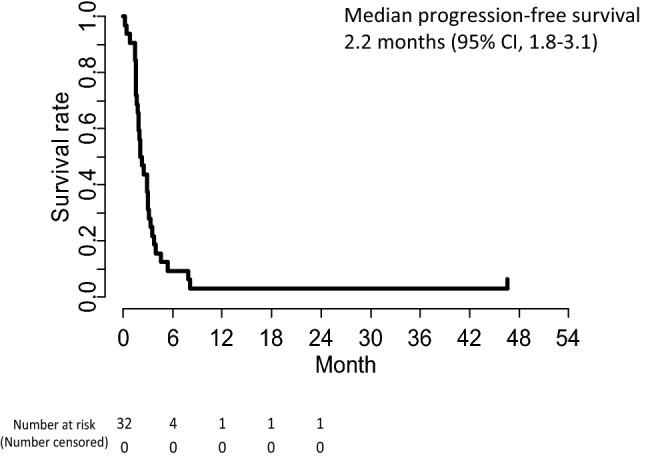
Kaplan–Meier curve of progression-free survival of patients with advanced or recurrent gastric cancer receiving tri-weekly nanoparticle albumin-bound paclitaxel (*nab*-PTX) at a dose of 220 mg/m^2^ in a second-line or later setting

**Fig. 2:**
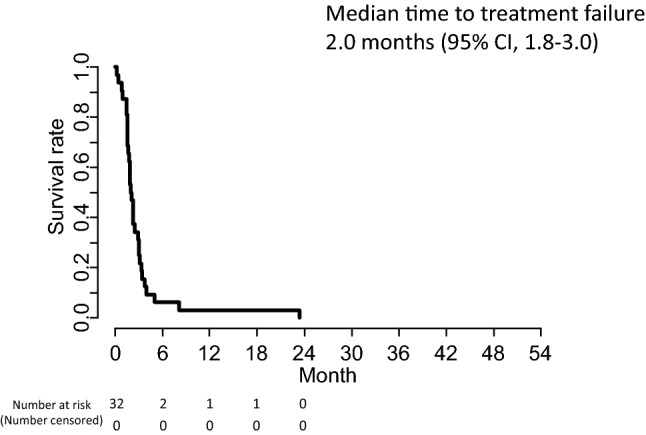
Kaplan–Meier curve of time to treatment failure of patients with advanced or recurrent gastric cancer receiving *nab*-PTX (220 mg/m^2^) in a second-line or later setting

**Fig. 3:**
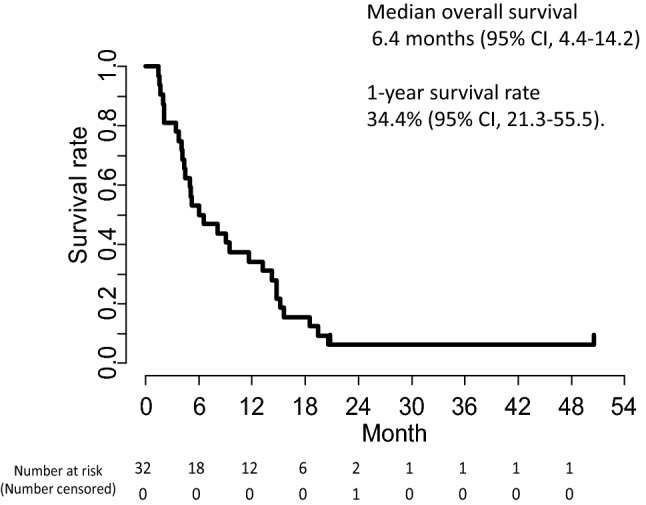
Kaplan–Meier curve of overall survival of patients with advanced or recurrent gastric cancer receiving *nab*-PTX in a second-line or later setting

The overall responses in the 32 patients are summarized in Table 2. Partial response was achieved in only one patient, yielding an overall RR of 3.1% (95% CI, 0–16.2%), which did not reach the protocol-specified threshold (*p* = 0.966). Stable disease (SD) was observed in 11 patients, providing a DCR of 37.5% (95% CI, 21.1–56.3%). At the data cut-off (February 2019), the median follow-up was 6.3 months, and the number of treatment courses administered ranged from 1 to 27, with a median of 3. Only 1 out of 32 patients increased their treatment dose to 260 mg/m^2^ in the second course. The median PFS was 2.2 months (95% CI, 1.8–3.1) with the 6-month PFS rate being 9.4% (95% CI, 3.2–27.5%; Fig. 1). The median TTF was 2.0 months (95% CI, 1.8–3.0, Fig. 2). The RDI was 97.8% (average dose of 215 mg/m^2^). The median OS was 6.4 months (95% CI, 4.4–14.2) and the 1-year survival rate was 34.4% (95% CI, 21.3%–55.5%: Fig. 3). Subsequent chemotherapy was received by 20 of the 32 patients (62.5%; Table 3), in which the most commonly selected regimen was CPT-11-based chemotherapy (60%).

**Table 2:** Clinical responses

	*n*	%	95% CI
Complete response (CR)	0	0	
Partial response (PR)	1	3.1	
Stable disease (SD)	11	34.4	
Progressive disease (PD)	16	50.0	
Not evaluated	4	12.5	
Response rate (RR)	1	3.1*	0.0–16.2%
Disease control rate (DCR): CR + PR + SD	12	37.5	21.1–56.3%

^*^p value for 10% threshold ratio: *p* = 0.966

**Table 3:** Subsequent chemotherapy after the study treatment

	*n* = 32
Any systemic therapy	20 (63%)
Number of regimens	
1	12 (38%)
2	6 (19%)
3	2 (6%)
Systemic anticancer agents	
CPT-11	12 (38%)
Ramucirumab	11 (34%)
Paclitaxel	4 (13%)
Oxaliplatin	4 (13%)
S-1	3 (9%)
Docetaxel	2 (6%)
Capecitabin	2 (6%)
Nivolumab	2 (6%)

Data are *n* (%)

### Toxicity

Grade 3 or 4 adverse events with an incidence rate of > 10% included neutropenia (38%), leucopenia (13%) and anemia (13%) as hematological toxicities, along with fatigue (19%), anorexia (16%), and CIPN (13%) as non-hematological toxicities. No patients experienced hypersensitivity or acute infusion reactions, although no premedication was administered at chemotherapy. Neither febrile neutropenia nor treatment-related deaths were observed (Table 4). The main reasons for treatment discontinuation or withdrawal were disease progression (26 cases: 81.3%) and adverse events (4 cases: 12.5%).

**Table 4:** Adverse events related to nanoparticle albumin-bound paclitaxel in patients with advanced or recurrent gastric cancer

	Grade				G1**–**4		G3**–**4	
	G1	G2	G3	G4	*n*	%	*n*	%
Hematological								
Leukopenia	5	5	2	2	14	43.8	4	12.5
Neutropenia	0	3	8	4	15	46.9	12	37.5
Anemia	6	5	4	0	15	46.9	4	12.5
Thrombocytopenia	4	0	1	0	5	15.6	1	3.1
Febrile neutropenia	0	0	1	1	1	3.1	1	3.1
Elevation of serum								
Creatinine	4	2	0	0	6	18.8	0	0.0
Total bilirubin	3	1	1	0	5	15.6	1	3.1
Aspartate aminotransferase	9	2	2	0	13	40.6	2	6.3
Alanine aminotransferase	5	3	1	0	9	28.1	1	3.1
Alkaline phosphatase	0	1	0	0	1	3.1	0	0.0
Hyponatremia	1	0	0	0	1	3.1	0	0.0
Hypoalbuminemia	4	2	3	0	9	28.1	3	9.4
Non-hematological								
Diarrhea	4	0	0	0	4	12.5	0	0.0
Stomatitis	1	3	1	0	5	15.6	1	3.1
Rash	2	2	1	0	5	15.6	1	3.1
Nausea	4	2	2	0	8	25.0	2	6.3
Vomiting	4	0	0	0	4	12.5	0	0.0
Fatigue	6	4	6	0	16	50.0	6	18.8
Anorexia	8	4	5	0	17	53.1	5	15.6
Peripheral neuropathy	9	7	4	0	20	62.5	4	12.5
Alopecia	7	12	0	0	19	59.4	0	0.0
Joint pain	3	0	0	0	3	9.4	0	0.0
Myalgia	1	0	0	0	1	3.1	0	0.0
Dyspnea	1	0	0	0	1	3.1	0	0.0
Limb/Trunk edema	1	1	0	0	2	6.2	0	0.0
Fever	2	1	0	0	3	9.4	0	0.0
Dysguesia	1	0	0	0	1	3.1	0	0.0

## Discussion

In patients with AGC, the efficacy of tri-weekly *nab*-PTX at 260 mg/m^2^ without anti-allergic premedication was demonstrated with an overall RR of 27.8% in a phase II trial in Japan [18]. However, relatively high toxicities, such as neutropenia and CIPN, were indicated, which was the limitation of the general use of *nab*-PTX for patients with AGC. Therefore, we investigated the safety and efficacy of a reduced dose (220 mg/m^2^) of tri-weekly *nab*-PTX for patients with AGC with PS-0, 1 plus 2 in a second-line or later setting.

At the time when the current study was ongoing, a result of the phase III ABSOLUTE study was reported [23], in which the survival benefits of tri-weekly *nab*-PTX (260 mg/m^2^) or weekly *nab*-PTX (100 mg/m^2^) were compared with weekly *sb*-PTX (80 mg/m^2^) in patients with previously treated AGC. The results showed that weekly *nab*-PTX showed non-inferiority to weekly *sb-*PTX, whereas tri-weekly *nab*-PTX failed to demonstrate non-inferiority to *sb-*PTX. Interestingly, the study also showed that weekly *nab*-PTX had considerably lower grade 3 or higher toxicity than tri-weekly *nab-*PTX especially in terms of neutropenia (41.1 *vs.* 64.8%) and CIPN (2.5 *vs.* 20.1%). As a result of these findings, tri-weekly *nab-*PTX (260 mg/m^2^) is not commonly utilized in a clinical setting in AGC.

In the current study, we observed grade 3 or higher neutropenia at a rate of 38%, febrile neutropenia at 3%, and grade 3 or higher CIPN at 13%, whereas previous trials in AGC evaluating tri-weekly nab-PTX at 260 mg/m^2^ showed grade 3 or higher neutropenia and CIPN at rates of 49.1–64.8% and 20.1–23.6%, respectively. These results suggest that tri-weekly *nab*-PTX at 220 mg/m^2^ achieved the expected reduction of adverse events, consistent with previous trials in MBC [21, 22]

With regard to the efficacy, however, RR, the primary endpoint of this study, was only 3.1%, in addition to the relatively short median PFS and OS of 2.2 and 6.3 months, respectively. In contrast, previous studies evaluating tri-weekly nab-PTX at 260 mg/m^2^ showed RR of 25–27.8% as well as PFS and OS of 2.9–3.8 months and 9.2–10.3 months, respectively [18, 23]. These data indicate that the efficacy of the reduced dose of *nab*-PTX is not satisfactory, unlike in MBC [14, 20], despite the considerably low toxicities, so it is not recommended in clinical practice. Potential reasons for the poorer efficacy in the current study than in previous ones in AGC [18, 23] include the difference in the treatment line (second line or later *vs.* exclusively second line) and the relatively poor patients background in our study. In comparison with the other studies, our study population tended to be older (median age, 70 *vs.* 63.5 and 66 years) and had poorer ECOG PS (PS-0, 56 vs. 58.9 and 69%; as shown in Table 5). Indeed, the RDIs of phase II study and ABSOLUTE study were 93.4% (243 mg/m^2^) and 88.06% (229 mg/m^2^), respectively, which were higher than that in our study (97.8%: 215 mg/m^2^). Furthermore, the poorer patients’ background might lead to lower frequency of subsequent chemotherapy in the current study (62.5%) vs. others (81.5% [18] and 71% [23]) as was the number of patients administered CPT-11-based chemotherapy as subsequent therapy (53.7% and 51% [18] vs. 37.5% [23]). These results may support the hypothesis that the insufficient efficacy in the current study was derived from the inadequate treatment dose and the lower rate of subsequent treatment that may due to the difference in baseline patient characteristics between this study and the others.

**Table 5:** Comparison of background factors and results

	Phase II study^18)^ (260 mg/m^2^)		Absolute study^23)^ (260 mg/m^2^)		Current study (220 mg/m^2^)	
	No. of patients	%	No. of patients	%	No. of patients	%
No. of patients	56		243		32	
Age						
Median (years)	63.5		66		70	
ECOG performance status						
0	33	58.9	167	69	18	56
1	23	41.1	72	30	12	38
2	0	0	4	2	2	6
No. of pretreatment regimens						
1	56	100	243	100	23	72
2	0	0	0	0	9	28
Subsequent treatment						
Any	44	81.5	165 (231)	71.4	20	62.5
CPT-11	29	53.7	124	51	12	37.5
ORR^a^ (CR + PR)	15	27.8	38 (150)	25	1	3.1
PFS^b^ (months)	2.9		3.8		2.2	
PDI^c^	243 mg/m^2^	93.4	229 mg/m^2^	88.06	215 mg/m^2^	97.8
Median duration of treatment (M: months)						
	2.6		2.4		2.0	
OS^d^ (months)	9.2		10.3		6.4	

(): The numbers in parentheses indicate the number of cases evaluated

^a^Overall response rate

^b^Progression-free survival (median)

^c^Relative dose intensity

^d^Overall survival (median)

In summary, the present study failed to show a clinical benefit of a reduced dose of tri-weekly *nab*-PTX in AGC and indicated that such treatment is not recommended, despite being potentially less toxic than the original dosing. Tri-weekly *nab-*PTX has little currency in the treatment of AGC as of the ABSOLUTE study [23], whereas the weekly administration of *nab-*PTX is now widely accepted. Following the success of the RAINBOW trial [7], weekly *nab*-PTX in combination with ramucirumab was examined in a phase II trial [24], showing promising activity with an RR of 54.8% and manageable toxicities. Given that the clinical benefit of weekly *nab*-PTX was pronounced in patients with peritoneal metastasis [23], a randomized phase II study comparing *sb-*PTX plus ramucirumab *with nab*-PTX plus ramucirumab is ongoing in AGC patients with peritoneal metastasis in a second-line setting (WJOG_P-SELECT study, jRCTs031180022), and the results of which are awaited.
